# The association between pre-existing cardiovascular disease and cancer treatment receipt in a population-based cancer registry

**DOI:** 10.1038/s41598-026-38529-0

**Published:** 2026-02-23

**Authors:** Hüseyin Küçükali, Gerard M. Walls, Damien Bennett, Anna Gavin, Mark Harbinson, Ciaran O’Neill

**Affiliations:** 1 Centre for Public Health, Queen’s University Belfast, Grosvenor Road, Belfast, BT12 6BA UK; 2 School of Medicine Dentistry and Biomedical Sciences, Queen’s University Belfast, Grosvenor Road, Belfast, BT12 6BA UK; 3 Research Center for Healthcare Systems and Policies, Istanbul Medipol University, Istanbul, Türkiye; 4 Cancer Centre Belfast City Hospital, Belfast Health and Social Care Trust, Belfast, UK; 5 Johnston Cancer Research Centre, Queen’s University Belfast, Belfast, UK; 6 Northern Ireland Cancer Registry, Queen’s University Belfast, Belfast, UK

**Keywords:** Cardio-oncology, Cardiotoxicity, Comorbidity, Multimorbidity, Cancer registration, Cardiology, Oncology, Cancer, Cardiovascular diseases, Comorbidities

## Abstract

Baseline cardiovascular disease (CVD) has been linked with poorer outcomes for patients with cancer. The precise mechanisms underpinning that are poorly understood but may include reduced treatment receipt. We estimated the association between pre-existing CVD and cancer treatment receipt using population-based cancer registry records. Records of all adults diagnosed with cancer (excluding non-melanoma skin cancer) in Northern Ireland in 2009–2019 were linked with comorbidity and treatment data. The adjusted odds ratios (aOR) of receiving various cancer treatments for patients with previous cardiovascular diagnoses were estimated using multivariable logistic regression, adjusting for established confounders. Subgroup analyses were conducted for combinations of 24 tumour sites, 11 cardiovascular conditions, and 5 cancer treatment modalities. Kaplan-Meier curves and Cox proportional hazards model were used to analyse time to treatment. 81,341 cancer patients were included, with a mean age of 67.1 ± 14.1 years. The most common cancers included were breast (15.8%), lung (14.1%) and colorectal cancer (13.5%). Patients with pre-existing CVD (23.4%) were 30% less likely to receive any cancer treatment than patients without (aOR = 0.70 [95%CI 0.67, 0.73]). This reduction varied between treatment modalities with 30% for chemotherapy (aOR = 0.70 [95%CI 0.67, 0.73]), 28% for radiotherapy (aOR = 0.72 [95%CI 0.66, 0.79]), and 23% for surgery (aOR = 0.77 [95%CI 0.74, 0.80]). Hormone therapy showed no significant overall difference (aOR = 1.02 [95%CI 0.94, 1.11]). At 6 months post-diagnosis, patients with pre-existing CVD had a 14% (0.14 [95%CI 0.13, 0.15]) lower probability of initiating cancer treatment compared to those without. Pre-existing CVD was associated with an overall lower odds of cancer treatment, although the magnitude of this decrement varied according to the primary tumour, treatment modality and the type of CVD. These data present granular population-based insights into the impact of cardiovascular comorbidities on receiving cancer treatment and should be accounted for when reporting survival variations and healthcare policymaking.

**Supplementary Information:**

The online version contains supplementary material available at 10.1038/s41598-026-38529-0.

## Background

While cardio-oncology remains a relatively new sub-speciality, its growth and evolution have been rapid^1^. Reasons postulated for this expansion include the ageing population, enhanced cancer survivorship focus, and a greater appreciation of the impact of some cancer therapies on cardiovascular risks^2,3^. Early contributions to this literature have been dedicated to scoping the landscape of this interdisciplinary field for key gaps in clinical practice. These gaps include the importance of predicting outcomes^4^ for cancer patients with pre-existing cardiovascular disease, in order to guide cardiac optimisation and monitoring. Equally, the resource implication of concomitant cardiovascular disease and cancer diagnoses for healthcare service and workforce planning, training and funding are important to understand so that care standards can be maintained and improved. Of importance for researchers and policymakers, is the opportunity to strengthen public health messaging around primary prevention by drawing attention to shared risk factors for cancer and CVD, especially where multiple relationships between shared modifiable risk factors have been illustrated^5^.

As the cardio-oncology subspecialty has matured, research has highlighted some potential to predict cardiotoxicity among those with existing CVD to inform patient management through, for example, consideration of alternative therapies or deployment of specific surveillance strategies during treatment^6–11^. Similarly, novel cardioprotective strategies with adjunct pharmacological therapies^12–15^, have shown promise in specific scenarios. Across the academic community, there is a consensus that further research is urgently required, and in particular real-world evidence, in order that disparities in treatment receipt can be better understood, in relation to cancer patients with pre-existing CVD^4^. Of note, much of the limited literature in this area originates from the United States, therefore real-world evidence from other populations with different health systems and ethnic compositions is urgently required.

Within this context, the investigators of this study sought to examine the relationships between pre-existing CVD and cancer treatment delivered in a population of cancer patients with access to publicly funded cancer and CVD care in Northern Ireland (NI), one of the devolved nations of the United Kingdom.

## Methods

### Study setting and data

This retrospective cohort study utilised nationally representative data from the Northern Ireland Cancer Registry (NICR) which is a population-based cancer registry.

NICR has collected cancer data for NI residents since 1993, using pathology reports, hospital records, and death registrations. 98.97% of cancer diagnoses were confirmed using histopathology, cytopathology, and hospital records, with only 1.02% based on death certificates alone^16^. Cancer Information Officers verify diagnoses following international standards, and can also use additional data from radiotherapy (ARIA), chemotherapy (RISOH) systems and multidisciplinary team discussions (Cancer Patient Pathway System) records. Further details on NICR’s data sources and data processing methodology, including the classification of cancer types, are available elsewhere^17^.

From 2017 to 2021, an average of 10,061 new cancer cases were recorded annually—an 8% rise from 2012 to 2016. The most common cancers were prostate, breast, lung, and colorectal. NICR is a population-based registry covering all 1.9 million NI residents. The population has grown 5% since 2011, with a median age of 39 and 18% aged 65+. NI is predominantly white population (96.6%), though ethnic diversity is rising. NI has universal healthcare which is funded by general taxation and free at point-of-use. Cancer incidence is 13% higher in the most deprived areas and 5% lower in the least deprived. Lung, head & neck, and cervical cancers are more common in deprived areas, while melanoma and prostate cancer are more common in less deprived areas^18^.

All patients aged 18 or older who were diagnosed with cancer, excluding non-melanoma skin cancer, for the first time in NI between 2009 and 2019 were included in the study. Using unique patient identifiers, the NICR securely and confidentially linked cancer records with other relevant centralised databases, including hospital admissions, systemic anticancer therapies, radiotherapy data, and prescriptions^19^.

### Outcome and exposure variables

The primary outcome of interest was the receipt of cancer treatments, including chemotherapy, hormone therapy, radiotherapy, and surgery. NICR have identified chemotherapy, hormone therapy and radiotherapy events from the Regional Information System for Oncology & Haematology (RISOH), Enhanced Prescribing Database (EPD) and regional radiotherapy information system (ARIA), respectively. Surgery data has been determined using OPCS Classification of Interventions and Procedures codes from the Patient Administration System (PAS)^20^. Hormone therapy records are only considered as cancer treatments for patients with hormone-sensitive tumours (breast, prostate and uterine). Radiotherapy data could only be reliably matched for 2018 and 2019 because the region-wide information system has provided unique patient identifiers from 2018 onwards.

The presence of a CVD diagnosis before or on the date of cancer diagnosis was the key exposure of interest. The definition of CVD included congestive heart failure (CHF), myocardial infarction, other ischaemic heart diseases (categorised together as angina), atrial fibrillation (AF), other cardiac arrhythmias, valvular disease, pulmonary circulation disorder, peripheral vascular disease (PVD), embolism and thrombosis, myopericarditis, and cardiac arrest. Data on cardiovascular diseases were derived from the diagnoses (including primary, subsidiary, and up to five secondary diagnoses) recorded at any hospital admission between 2006 and 2019 using ICD-10 codes as previously reported^21^.

### Confounder variables

**Fig. 1:**
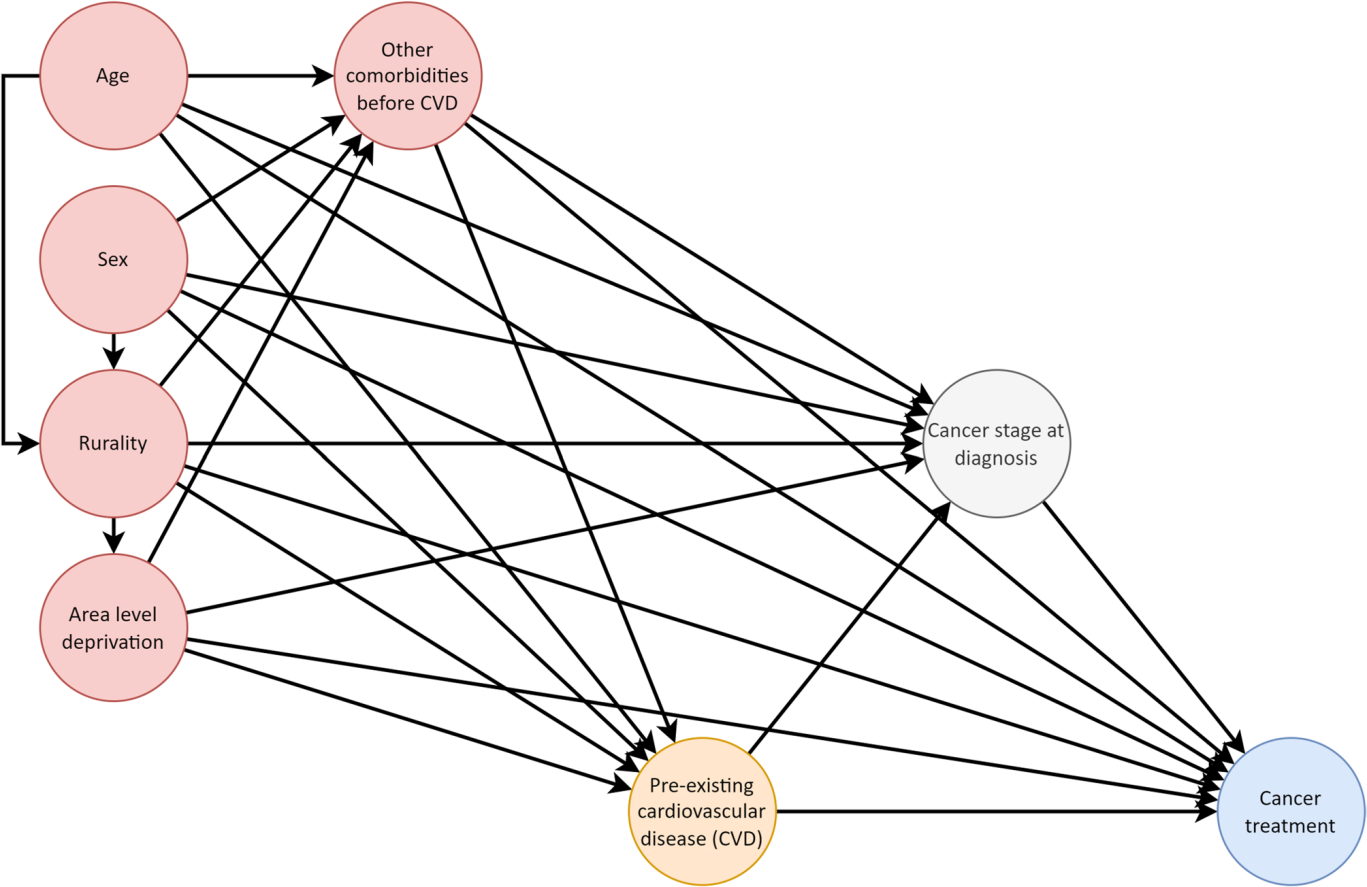
Causal assumptions for the effect of pre-existing cardiovascular diseases on receiving cancer treatment. Other diseases are represented as a single node for simplicity, but we considered the following disease groups separately: cerebrovascular disease, chronic pulmonary disease, hypertension, diabetes, liver disease, renal disease, peptic ulcer, anaemia, neurodegenerative disorders, rheumatoid disorders. Also, while analysing the effect of a specific CVD in subgroup analyses, other CVDs are also considered as comorbidities. The graph is available at https://opencausal.org/graph/brsnb5u5.

A directed acyclic graph (Fig. 1) visualises causal assumptions on the relationship between pre-existing CVD and receipt of cancer treatments^22^. Potential confounders were identified based on domain expertise as follows: age, sex, deprivation, rurality and other comorbidities. The cancer stage at the diagnosis was not considered a confounder, but instead a mediator in this relationship. By definition, a confounder must precede both exposure and outcome and not be an intermediate in the causal pathway. Although cancer stage can and does influence the availability of cancer treatment options, it can be influenced by pre-existing CVD. For example, patients with pre-existing CVD could be diagnosed at an earlier stage of cancer due to more frequent healthcare contact or closer examinations. Deprivation was measured using the NI Multiple Deprivation Measure^23^ and categorised by quintiles. Rurality consisted of three categories: urban, mixed, and rural^24^. Both deprivation and rurality were at the area level (i.e. Super Output Area), based on where the patient resided at the time of cancer diagnosis. Other comorbidities included cerebrovascular disease, chronic pulmonary disease, hypertension, diabetes, liver disease, renal disease, peptic ulcer, anaemia, neurodegenerative disorders, and rheumatoid disorders. Similar to cardiovascular diseases, other comorbidities were identified from the diagnoses at hospital admissions in 2006–2019 using ICD-10 codes as previously reported^21^.

A complete list and description of all variables used in the analyses, as well as ICD-10 codes used to define cardiovascular diseases and other comorbidities, are provided in Supplemental Appendix 1 (Tables S1.1–3).

### Statistical analysis

To describe the study sample, counts and percentages were calculated for all variables except that age was summarised with mean and standard deviation. Distributions of variables were compared between groups with and without pre-existing CVD using the chi-squared test and t-test for categorical and continuous variables, respectively.

The adjusted odds ratio (aOR) of receiving cancer treatment for those with pre-existing CVD compared to those without was estimated using multivariable logistic regression analysis. Age and deprivation deciles were normalised (min-max) before inclusion in the model. Based on the same assumptions, aORs were estimated for each treatment modality via separate models. Heteroscedasticity-consistent (HC3) standard error estimators were used. As treatment data were not available for the entire study period for all modalities, the analysis was limited to the subsets of patients whose cancer diagnosis date was within the range of data availability. Model covariates were selected a priori based on domain expertise and the directed acyclic graph. We used the back-door criteria to identify a minimally sufficient adjustment set to control for confounding. The adjustment set included age, sex, deprivation, geographical rurality and non-cardiovascular comorbidity.

To account for the heterogeneity that exists between patients with different cancers and specific types of treatment, we examined the association between CVD and odds of treatment with granular patient subgroups based on primary tumour site, specific cardiovascular comorbidities and cancer treatment modalities. For subgroup analysis, a separate multivariable logistic regression model was built for each combination of 24 tumour sites, 11 CVD conditions and up to 4 relevant treatment modalities. When modelling the association between a specific CVD subtype and each cancer treatment, CVD conditions other than the CVD of interest were also considered as confounders. Sensitivity analyses explored different model specifications and generational changes by year of diagnosis.

To account for deaths and loss to follow-up, we also analysed the time to cancer treatment since their cancer diagnosis. Time was calculated in days from the initial cancer diagnosis to the start of any cancer treatment (surgery, chemotherapy, hormone therapy, radiotherapy), death, loss to follow-up, or the end of the study (December 31, 2019), whichever occurred first. Kaplan-Meier methods were used to estimate the cumulative probability of treatment initiation over time. We compared the treatment probability of patients with and without pre-existing CVD by Risk Difference and Risk Ratios at 6 and 12 months. Confidence intervals were calculated using the delta method. Further, we used a multivariable Cox model to estimate hazard ratios (HRs) for treatment receipt, adjusting for identified confounders.

Data analysis was programmed in Python using the statsmodels (0.14.5) and scipy (1.16.1), lifelines (0.30.0) packages. The analysis code is provided online^25^.

## Results

**Fig. 2:**
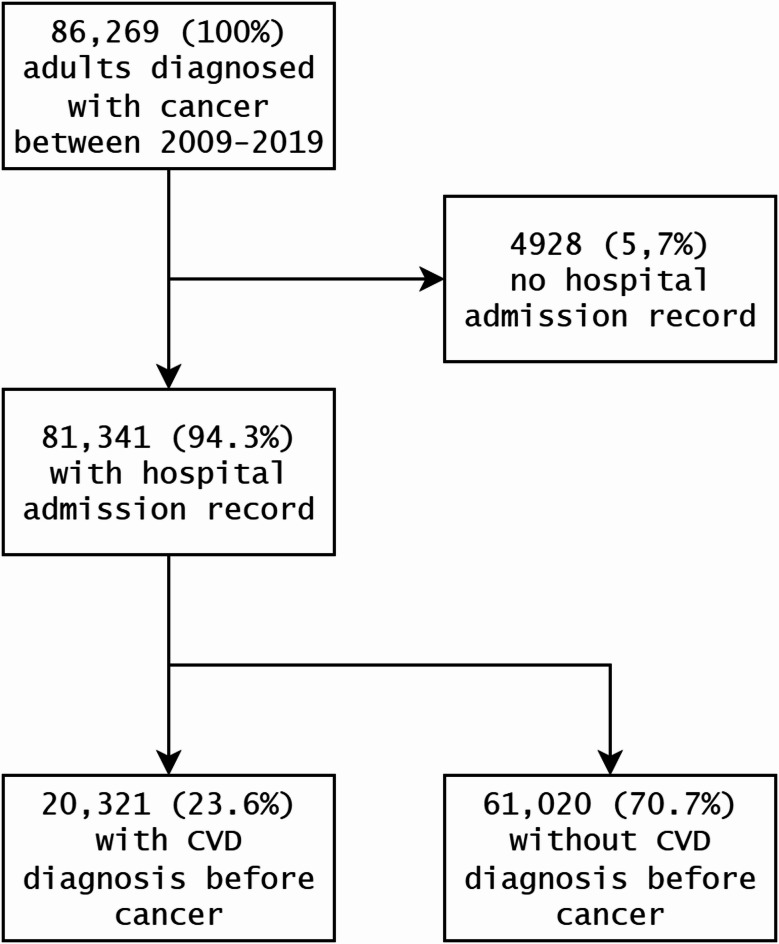
Breakdown of the number of cancer patients included in the study by cardiovascular disease onset.

A total of 86,269 adults were diagnosed with cancer between 2009 and 2019, for which comorbidity data were available for 81,341 (94.2%) (Fig. 2). A quarter of these patients (25%) were diagnosed with CVD prior to their cancer diagnosis. Patient characteristics are shown in Table 1. A total of 50% of included patients were female, the mean age was 67.1 (SD:14.1) years at the time of cancer diagnosis, and 59.9% were residents in an urban area. The most common tumour sites were breast (15.8%), lung (14.1%), and colorectal cancer (13.5%), and the most prevalent cardiovascular diseases were other ischaemic heart diseases (12.9%), atrial fibrillation (8.0%), and myocardial infarction (5.5%). Distributions of the patients by all tumour and cardiovascular disease types are provided in Supplemental Appendix 1 (Tables S1.4–5).

**Table 1:** Characteristics of the study sample.

	Total	Pre-existing CVD	No pre-existing CVD	*p*
Age	67.11 ± 14.10	75.31 ± 10.42	64.37 ± 14.11	< 0.001
Gender				
Male	40,646 (50.0%)	12,200 (60.0%)	28,446 (46.6%)	< 0.001
Female	40,695 (50.0%)	8121 (40.0%)	32,574 (53.4%)	
Residence				
Urban	48,717 (59.9%)	12,251 (60.3%)	36,466 (59.8%)	0.280
Rural	25,523 (31.4%)	6343 (31.2%)	19,180 (31.4%)	
Mixed urban/rural	7098 (8.7%)	1727 (8.5%)	5371 (8.8%)	
Deprivation Quintile				
1 st (Most Deprived)	15,352 (18.9%)	3952 (19.4%)	11,400 (18.7%)	< 0.001
2nd	16,931 (20.8%)	4395 (21.6%)	12,536 (20.5%)	
3rd	16,608 (20.4%)	4228 (20.8%)	12,380 (20.3%)	
4th	16,563 (20.4%)	4070 (20.0%)	12,493 (20.5%)	
5th (Least Deprived)	15,884 (19.5%)	3676 (18.1%)	12,208 (20.0%)	
Stage				
1	20,755 (31.9%)	3999 (27.4%)	16,756 (33.2%)	< 0.001
2	14,482 (22.2%)	2889 (19.8%)	11,593 (22.9%)	
3	13,512 (20.7%)	3108 (21.3%)	10,404 (20.6%)	
4	16,377 (25.1%)	4613 (31.6%)	11,764 (23.3%)	
Cerebrovascular Disease				
No	78,643 (96.7%)	18,513 (91.1%)	60,130 (98.5%)	< 0.001
Yes	2698 (3.3%)	1808 (8.9%)	890 (1.5%)	
Chronic Pulmonary Disease				
No	75,493 (92.8%)	16,818 (82.8%)	58,675 (96.2%)	< 0.001
Yes	5848 (7.2%)	3503 (17.2%)	2345 (3.8%)	
Hypertension				
No	67,761 (83.3%)	11,806 (58.1%)	55,955 (91.7%)	< 0.001
Yes	13,580 (16.7%)	8515 (41.9%)	5065 (8.3%)	
Diabetes				
No	76,063 (93.5%)	16,962 (83.5%)	59,101 (96.9%)	< 0.001
Yes	5278 (6.5%)	3359 (16.5%)	1919 (3.1%)	
Liver Disease				
No	80,690 (99.2%)	19,994 (98.4%)	60,696 (99.5%)	< 0.001
Yes	651 (0.8%)	327 (1.6%)	324 (0.5%)	
Renal Disease				
No	78,744 (96.8%)	18,926 (93.1%)	59,818 (98.0%)	< 0.001
Yes	2597 (3.2%)	1395 (6.9%)	1202 (2.0%)	
Peptic Ulcer				
No	80,555 (99.0%)	19,882 (97.8%)	60,673 (99.4%)	< 0.001
Yes	786 (1.0%)	439 (2.2%)	347 (0.6%)	
Anaemia				
No	79,753 (98.0%)	19,463 (95.8%)	60,290 (98.8%)	< 0.001
Yes	1588 (2.0%)	858 (4.2%)	730 (1.2%)	
Neurodegenerative Disorders				
No	79,411 (97.6%)	19,233 (94.6%)	60,178 (98.6%)	< 0.001
Yes	1930 (2.4%)	1088 (5.4%)	842 (1.4%)	
Rheumatoid Disorders				
No	80,354 (98.8%)	19,761 (97.2%)	60,593 (99.3%)	< 0.001
Yes	987 (1.2%)	560 (2.8%)	427 (0.7%)	

The odds of receiving any cancer treatment were 30% lower (aOR = 0.70 [95%CI 0.67,0.73]) in patients who had pre-existing CVD after adjusting for age, sex, deprivation, rurality, and other comorbidities. This was fairly consistent for chemotherapy 30% and radiotherapy 28%, but the magnitude was slightly lower for surgery at 23%. Having only patients with hormone-sensitive tumours (breast, prostate, and uterine) included, the analysis did not show a significant overall difference for hormone therapy. Adjusted odds ratios with accompanying confidence intervals are shown in Table 2. Full regression results are provided in Supplemental Appendix 2 (Tables S2.1–5).

**Table 2:** Adjusted odds ratios for receiving cancer treatment for cancer patients with pre-existing cardiovascular disease.

	aOR [95%CI]	Data availability	Sample
Any Treatment	0.70 [0.67, 0.73]	2009–2019	81,341
Chemotherapy	0.70 [0.67, 0.73]	2009–2019	81,341
Hormone Therapy	1.02 [0.94, 1.11]	2010-2019^a^	23,649^c^
Radiotherapy	0.72 [0.66, 0.79]	2018–2019	16,043
Surgery	0.77 [0.74, 0.80]	2009-2019^b^	81,341

*aOR* adjusted Odds Ratio, *95%CI* 95% Confidence Intervals. Each adjusted odds ratio is estimated by a separate logistic regression model which is adjusted for confounders including age, sex, deprivation, rurality and other comorbidities. Only patients diagnosed with cancer within the period that treatment data is available are included in the analyses. ^a^One patient was treated in the last days of 2009. ^b^Two patients were treated in the last days of 2008. ^c^Only patients with breast, prostate and uterine cancer were included.

**Fig. 3:**
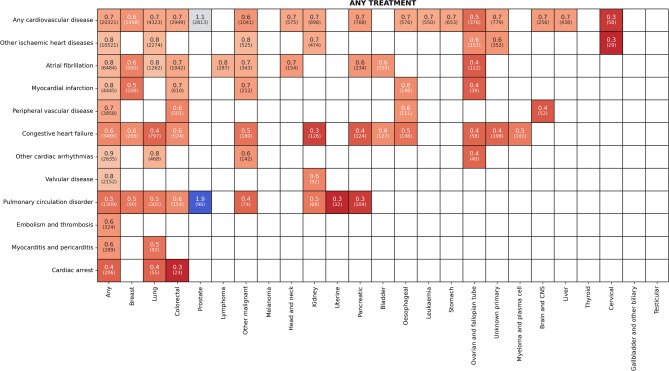
Adjusted odds ratios for receiving any cancer treatment for cancer patients with pre-existing CVD by tumour sites and cardiovascular disease types. Each adjusted odds ratio is estimated by a separate logistic regression model which is adjusted for confounders including age, sex, deprivation, rurality and other comorbidities. The number of patients with given Cancer-CVD combinations is provided in parentheses. Red corresponds to a negative effect (less likely to have treatment) and blue to a positive (more likely to have treatment), with darker shades indicating greater effect size. Values with confidence intervals containing null are omitted here for visual clarity. A detailed table of estimates with corresponding confidence intervals can be found at osf.io/vgzq5.

The results of subgroup analyses are summarised in Fig. 3, and further subgroups by treatment modalities are included in Supplemental Appendix 3 (Figures S3.1–4). Notably, the odds of receiving some treatments was less than 0.5 in the presence of pre-existing CVD across several cancers. The most common cancer types were not spared treatment receipt heterogeneity according to baseline CVD status. For example, in patients with breast cancer, pre-existing CHF was associated with a 40% lower odds of receiving any treatment, and AF, a 45% lower odds. In patients with colorectal cancer, CHF was associated with 41% lower treatment receipt, whereas AF was associated with 28%. In patients with lung cancer, while CHF was associated with 56% lower treatment receipt, it was only 16% for AF. Some unexpected positive associations were also identified in the data. For example, patients with prostate cancer with pre-existing CVD were 12% more likely to receive any treatments than those without. The results of subgroup analyses with confidence intervals and the number of patients are provided online^25^.

Sensitivity analyses showed similar results. Different model specifications yielded ORs ranging from 0.43 [95%CI 0.41, 0.44] in the unadjusted model to 0.76 [95%CI 0.72, 0.79] in a model over-adjusted for the cancer stage at diagnosis in addition to identified confounders. Although there were fluctuations in OR for receiving treatment by the year of cancer diagnosis, no particular trend was observed for any treatment modalities. Details of the sensitivity analyses are provided in Supplemental Appendix 4 (Tables S4.1–4, Figure S4.1).

**Table 3:** Comparison of cumulative probabilities of initiation of cancer treatment at 6 and 12 months post-diagnosis for cancer patients with and without pre-existing CVD.

Groups	Cumulative probability of treatment [95%CI]	
	At 180 days	At 365 days
Pre-existing CVD	0.63 [0.62, 0.64]	0.66 [0.66, 0.67]
No pre-existing CVD	0.77 [0.77, 0.78]	0.80 [0.79, 0.80]
*Risk Difference*	−0.14 [−0.15, −0.13]	−0.13 [−0.14, −0.13]
*Risk Ratio*	0.82 [0.81, 0.83]	0.83 [0.82, 0.84]

**Fig. 4:**
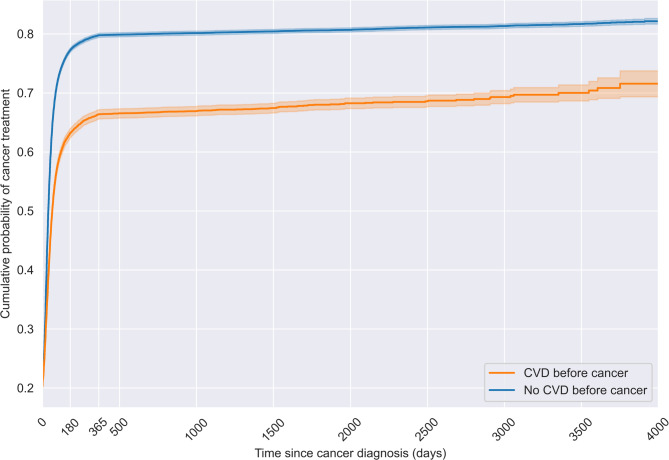
Kaplan–Meier cumulative probability of cancer treatment over time since cancer diagnosis for patients with and without pre-existing CVD.

Kaplan–Meier curves in Fig. 4 revealed early and persistent divergence in treatment probability between the two groups. Table 3 shows the cumulative probabilities at 6 and 12 months and related risk calculations. Cancer patients with pre-existing CVD had a lower cumulative probability of cancer treatment by 6 months post-diagnosis (0.63 [0.62, 0.64]) compared to patients without (0.77 [0.77, 0.78]). The absolute risk difference was − 0.14 [−0.15, −0.13] and the risk ratio was 0.82 [0.81, 0.83], indicating a substantial treatment delay among those with pre-existing CVD. In the Cox model, pre-existing CVD was associated with a lower *hazard* of cancer treatment receipt (HR = 0.86 [95%CI 0.84, 0.88]), even after accounting for sociodemographic variables and other major comorbidities. This suggests that CVD independently contributes to delayed treatment uptake. Full model results are provided in Supplemental Appendix 2 (Table S2.6).

## Discussion

Real-world population-based evidence in examining the relationship between CVD, cancer and outcomes has been identified as a key gap in the cardio-oncology literature^4^. This need extends to the likelihood of receiving treatment, which is crucial to understanding relationships with outcomes including survival. This study offers valuable insights from a health system where financial security and health insurance factors are less relevant than in data from the United States.

This study uses a large population-based cohort from NI with access to publicly-funded healthcare. Considering over 80,000 patients in a jurisdiction with a population of 1.9 million this study offers comprehensive and unique population-level insights. The results clearly show that pre-existing CVD is significantly associated with the odds of receiving cancer treatment and time to treatment initiation, which builds on previous work undertaken by the investigators on reduced all-cause and cancer-specific survival for cancer patients with pre-existing CVD^21^. In the current study, pre-existing CVD was associated with a lower odds (23–30%) of receiving cancer treatment across many subtypes of CVD, cancer and treatment. Findings also indicate that cancer patients with pre-existing CVD are 14% less likely to receive treatment within 6 months of cancer diagnosis compared to those without CVD, showing a potentially important disparity in cancer care delivery. The observed association persisted after adjusting for sociodemographic factors and other major comorbidities, suggesting that pre-existing CVD may play an independent role in delaying cancer treatment. These associations may at least partially explain the survival penalty demonstrated in the previous study^21^. Taken together with other data, this study indicates that cardiovascular comorbidity should be a core consideration for future international comparisons of cancer survival outcomes.

The granularity of the presented analysis in terms of primary cancer and CVD subtypes enabled important heterogeneity to be elucidated. Figure 3 summarises how pre-existing CVD is associated with the odds of treatment, depending on the specific cancer and CVD condition, and the specific treatments concerned. For example, CHF was shown to be associated with significantly reduced odds of receiving any cancer treatment ranging from a 40% to 67% reduction. For such at-risk clinical subgroup scenarios, optimisation of available treatments, additional cardiac-centric support during treatment, and bespoke follow-up strategies are therefore warranted. Interestingly, patients with AF, were only 16% to 59% less likely, and patients with PVD were 29% to 60% less likely, to receive any cancer treatment. This suggests that CVD conditions which are perceived by surgeons and oncologists to be more manageable may influence the treatment receipt less.

Pre-existing CVD not only makes certain modalities less favourable but also reduces the odds of receiving any treatment and delays the treatment initiation. This study is the first attempt to quantify this treatment deficit, which has been observed in clinical practice to some extent. Further studies should elaborate on specific reasons for reduced and delayed treatment receipt in different cancer types.

There are several important underpinning factors that govern the probability of a patient receiving oncology treatments beyond those included in the analysis. It is worth noting that treatment modalities vary by cancer stage, e.g. hormone therapies are widely used for prostate cancer whereas chemotherapy is used less often and mostly for late-stage disease. Radiotherapy is mostly used for localised disease, but is also a useful treatment for relieving symptoms from metastases. These radiotherapy approaches vary considerably in terms of the resource required, side effect profile and anatomical relationship to the heart, although this level of data was not available. Furthermore, patients with CVD might have different likelihoods of certain treatment options secondary to overlapping symptoms that delay the cancer diagnosis, which could be directly impacted by their cardiovascular history. For example, clinicians caring for patients with CHF might miss cardinal features of lung cancer because many of these overlap with symptoms and clinical signs of CHF. Alternatively, as described above, additional monitoring may lead to earlier detection of some cancers, e.g. patients getting regular electrocardiograms for arrhythmia may have a melanoma on the torso noticed earlier than they otherwise would have. Further work focused on specific cancers could, with additional data, help parse these relationships examining, for example, deployment and timing of specific regimens for specific cardiovascular comorbidities.

Our interdisciplinary analysis highlights the value of examining heterogeneity related to different primary cancer and CVD subtypes in large databases. Cancer treatment decisions are typically tailored based on these factors and diagnostic and prehabilitation pathways have the potential to incorporate these factors. In a number of instances, the variation in treatment receipt observed was clinically logical and entirely warranted. For example, individuals with CHF receive less high-risk cancer treatment across a range of cancer types, but thromboembolic disease does not have this association, since safe, effective and inexpensive options exist to address/pre-empt the complications. Certain types of chemotherapy might be more likely to damage the heart while this is not true for hormonal therapy, and therefore a pre-existing cardiac problem may influence a decision regarding chemotherapy more than a decision regarding hormone treatment. In other CVD types however, a direct relationship may not be so clear, as shown by the heterogeneity for the CVD subtype, arrhythmia.

As showcased by this study, detailed real-world evidence in understanding differences in receiving treatment has the potential to address nuanced, clinically important, cardio-oncology questions. The relationships explicated here and with respect to survival^21^ could significantly strengthen public health messaging around shared risk factors for CVD and cancer, for example with respect to smoking, exercise and diet. The findings may also help explain variations between countries in cancer statistics, including outcomes especially where survival for the UK is lower than many similar countries^26^.

In terms of the strengths of this study, it represents a population-based analysis within a publicly funded system with accurate and complete cancer registration system^16^ and so issues of sample selection or where access to care may be predicated on factors other than need, are less likely to arise. Also, the analysis offers favourable degree of granularity for important confounding factors such as age, sex, deprivation, rurality and other comorbidities. These data support other recent tumour-specific studies in the field which demonstrate how cardiovascular health is an important factor in oncological decision making^27–29^.

Beyond the limits of retrospective analyses, in terms of weaknesses, cardiovascular baseline status was defined according to secondary care documentation, and therefore may be under-reported for patients that did not require admission in the years preceding their cancer diagnosis. Cancer treatment was not linked to cancer diagnoses, so for the very small numbers of patients with a second cancer diagnosis, treatments possibly could be related to the subsequent cancer. Additionally, some treatment modalities were not part of the standard care for some tumour sites yet still included in the analyses for completeness and consistency. Although the four major modalities were analysed, immunotherapy, targeted, and monoclonal antibody therapies were omitted because data were available to us for only a small number of patients for these therapies. We did not examine the specific treatments within treatment groups – for example, known cardiotoxic drugs^30^ or thoracic radiotherapy doses to the heart^31^. Similarly, comorbidity data was limited to diagnostic codes and it did not allow us to assess the severity or temporal proximity to cancer diagnosis of pre-existing CVDs, which may influence treatment decisions. Because we had data on administered treatments instead of planned treatments, and so cancer patients died before being able to receive a treatment are counted as not received treatment. Future studies should explore these issues where data availability permits. Lastly, logistic regression models may overestimate the effect when the event (or non-event) is not rare, which could be a particular concern for subgroups with moderate levels of treatment receipt. We employed additional statistical approaches, time-to-event models, to triangulate our findings.

## Conclusions

Pre-existing CVD was found to be associated with a significant reduction in the odds of receiving, and a delay in, common cancer treatments in a publicly funded healthcare system. The reduction in treatment receipt depended on cancer, cancer treatment and CVD type. This study provides population-based evidence for different subgroups and emphasises the necessity of considering heterogeneity. A reduced treatment receipt for some subgroups may be expected because cancer treatment decisions are appropriately adapted according to the CVD history of patients. However, unexpected reductions require closer investigation to ensure treatment decisions are in the best interest of patients and that potential underlying access issues or biases in care provision are identified and addressed. That receipt of treatments is related to pre-existing CVD underscores the importance of assessing and addressing potentially modifiable risks. The impact of comorbidities on cancer outcomes should be a core consideration for future international comparisons of cancer survival outcomes.

## Supplementary Information

Below is the link to the electronic supplementary material.

Supplementary Material 1

## Data Availability

The datasets generated and/or analysed as part of this study are not publicly available due to the limits of the ethical approval granted to the Northern Ireland Cancer Registry to share patient-level data. Anonymised, non-patient level data can be made available from the corresponding author on reasonable request.
