# Supplementary material for: The association between pre-existing cardiovascular disease and cancer treatment receipt in a population-based cancer registry
Source: Sci Rep. 2026 Feb 23;16:10232. doi: 10.1038/s41598-026-38529-0 (PMC13031606; doi:10.1038/s41598-026-38529-0)
Supplement: Supplementary file 1 — Supplementary Material 1 [file 41598_2026_38529_MOESM1_ESM.pdf]

Supplemental Appendix 1. Variable Definitions and Disease Frequencies

Variable Definitions

Table S1.1. Variable definitions

Variable	Description	Values
Treatment receipt:<br>• Chemotherapy<br>• Hormone therapy<br>• Radiotherapy<br>• Surgery<br>• Any treatment	The existence of chemotherapy, hormone therapy and radiotherapy events from the Regional Information System for Oncology & Haematology (RISOH), Enhanced Prescribing Database (EPD) and regional radiotherapy information system (ARIA), respectively. Surgery data has been determined using OPCS codes from the Patient Administration System (PAS). [1]	Categorical<br><br>0: Not received<br>1: Received
Pre-existing CVD:<br>• Other ischaemic heart diseases (excluding myocardial infarction)<br>• Atrial fibrillation<br>• Myocardial infarction<br>• Peripheral vascular disease<br>• Congestive heart failure<br>• Other cardiac arrhythmias (excluding atrial fibrillation)<br>• Valvular disease<br>• Pulmonary circulation disorder<br>• Embolism and thrombosis<br>• Myocarditis and pericarditis<br>• Cardiac arrest<br>• Any cardiovascular disease	The existence of diagnosis of the cardiovascular disease in the PAS before the cancer diagnosis.<br><br>See Table S1.2 for ICD-10 codes used to define CVDs.	Categorical<br><br>0: No pre-existing CVD<br>1: Pre-existing CVD
Age	Age in years at cancer diagnosis.	Continuous<br><br>Normalized (min-max) for regression models
Sex	Registered sex.	Categorical<br><br>0: Female<br>1: Male
Rurality	Whether the patient resided in an area (Super Output Area) categorized	Categorical<br><br>0: Urban or mixed

	as rural at the time of cancer diagnosis. [2]	1: Rural
Deprivation	Deprivation index (ranging from 0 to 1) of the area (Super Output Area) where the patient resided at the time of cancer diagnosis, based on the Northern Ireland Multiple Deprivation Measure. [3]	Continuous
Co-morbidities:<br>• Cerebrovascular disease<br>• Chronic pulmonary disease<br>• Hypertension<br>• Diabetes<br>• Liver disease<br>• Renal disease<br>• Peptic ulcer<br>• Anaemia<br>• Neurodegenerative disorders<br>• Rheumatoid disorders	The existence of diagnosis of the disease in the PAS before the cardiovascular disease diagnosis.<br><br>See Table S1.3 for ICD-10 codes used to define other comorbidities.	Categorical<br><br>0: Not-diagnosed<br>1: Diagnosed
Tumour stage	TNM stage at the time of cancer diagnosis.	Continuous
Time to treatment	Time in days from the initial cancer diagnosis to the start of any cancer treatment (surgery, chemotherapy, hormone therapy, radiotherapy), death, loss to follow-up, or the end of the study (December 31, 2019), whichever occurred first.	Continuous

Table S1.2. ICD-10 codes used to define cardiovascular diseases [4]

**Cardiovascular Diseases**	**ICD-10 Codes**
Other ischaemic heart diseases (excluding myocardial infarction)	I20.x, I25.0, I25.1, I25.3, I25.4, I25.5, I25.6, I25.8, I25.9
Atrial fibrillation	I48.x
Myocardial infarction	I21.x, I22.x, I25.2
Peripheral vascular disease	I67.x, I70.x, I71.x, I73.1, I73.8, I73.9, I77.1, I79.0, I79.2, K55.1, K55.8, K55.9, Z95.8, Z95.9
Congestive heart failure	I09.9, I11.0, I13.0, I13.2, I25.5, I42.0, I42.5, I42.6, I42.7, I42.8, I42.9, I43.x, I50.x, P29.0
Other cardiac arrhythmias (excluding atrial fibrillation)	I44.1, I44.2, I44.3, I45.6, I45.9, I47.x, I49.x, R00.0, R00.1, R00.8, T82.1, Z45.0, Z95.0
Valvular disease	A52.0, I05.x, I09.1, I09.8, I34.x, I35.x, I36.x, I37.x, I38.x, I39.x, Q23.0, Q23.1, Q23.2, Q23.3, Z95.2, Z95.3, Z95.4
Pulmonary circulation disorder	I26.x, I27.x, I28.0, I28.8, I28.9
Embolism and thrombosis	I74.x, I82.x
Myocarditis and pericarditis	I30.x, I31.x, I32.x, I40.x, I41.x
Cardiac arrest	I46.x
Any cardiovascular disease	Having one or more of the above

Table S1.3. ICD-10 codes used to define other comorbidities [4]

Comorbidities	ICD-10 Codes
Cerebrovascular disease	G45.x, G46.x, H34.0, I60.x, I61.x, I62.x, I63.x, I64.x, I65.x, I66.x, I67.x, I68.x, I69.x
Chronic pulmonary disease	I27.8, I27.9, J40.x, J41.x, J42.x, J43.x, J44.x, J45.x, J46.x, J47.x, J60.x, J61.x, J62.x, J63.x, J64.x, J65.x, J66.x, J67.x, J68.4, J70.1, J70.3
Hypertension	I10.x, I11.x, I12.x, I13.x, I15.x
Diabetes	E10.x, E11.x, E12.x, E13.x, E14.x
Liver disease	B18.x, I85.0, I85.9, I86.4, I98.2, K70.0, K70.1, K70.2, K70.3, K70.4, K70.9, K71.1, K72.1, K71.3, K71.4, K71.5, K71.7, K72.9, K73.x, K74.x, K76.0, K76.2, K76.3, K76.4, K76.5, K76.6, K76.7, K76.8, K76.9, Z94.4
Renal disease	I12.0, I13.1, N03.2, N03.3, N03.4, N03.5, N03.6, N03.7, N05.2, N05.3, N05.4, N05.5, N05.6, N05.7, N18.x, N19.x, N25.0, Z49.0, Z49.1, Z49.2, Z94.0, Z99.2
Peptic ulcer	K25.x, K26.x, K27.x, K28.x
Anaemia	D50.x, D51.x, D52.x, D53.x, D62.x
Neurodegenerative disorders	F00.x, F01.x, F02.x, F03.x, F05.1, G10.x, G11.0, G11.1, G11.2, G11.3, G11.8, G11.9, G12.x, G13.x, G20.x, G22.x, G25.4, G25.5, G30.x, G31.1, G31.2, G31.8, G31.9, G32.x, G35.x, G37.x, G40.x, G41.x, G93.1, G93.4, R47.0, R56.x
Rheumatoid disorders	L94.0, L94.1, L94.3, M05.x, M06.x, M08.x, M12.0, M12.3, M30.x, M31.0, M31.1, M31.2, M31.3, M32.x, M33.x, M34.x, "M35.x, M45.x, M46.1, M46.8, M46.9

Disease Frequencies

Table S1.4. Distribution of patients by tumour types

Tumour Site	n	%
Breast cancer	12848	15.8%
Lung cancer	11489	14.1%
Colorectal cancer	10950	13.5%
Prostate cancer	10766	13.2%
Lymphoma	3757	4.6%
Other malignant cancer	3643	4.5%
Melanoma	2988	3.7%
Head and neck cancer	2874	3.5%
Kidney cancer	2764	3.4%
Uterine cancer	2396	2.9%
Pancreatic cancer	2248	2.8%
Bladder cancer	2120	2.6%
Oesophageal cancer	2023	2.5%
Leukaemia	2013	2.5%
Stomach cancer	1986	2.4%
Ovarian and fallopian tube cancer	1944	2.4%
Unknown primary cancer	1831	2.3%
Myeloma and plasma cell neoplasms	1414	1.7%
Brain and central nervous system cancer	1253	1.5%
Liver cancer	1109	1.4%
Thyroid cancer	900	1.1%
Cervical cancer	848	1.0%
Gallbladder and other biliary cancer	791	1.0%
Testicular cancer	642	0.8%
Any cancer	81341	100.0%

Table S1.5. Distribution of patients by pre-existing cardiovascular disease types

Cardiovascular Diseases	n	%
Other ischaemic heart diseases	10521	12.9%
Atrial fibrillation	6484	8.0%
Myocardial infarction	4445	5.5%
Peripheral vascular disease	3858	4.7%
Congestive heart failure	3486	4.3%
Other cardiac arrhythmias	2635	3.2%
Valvular disease	2152	2.6%
Pulmonary circulation disorder	1309	1.6%
Embolism and thrombosis	324	0.4%
Myocarditis and pericarditis	289	0.4%
Cardiac arrest	206	0.3%
Any cardiovascular disease	20321	25.0%
No pre-existing CVD	61020	75.0%
Total	81341	100.0%

References

1. *Cancer Incidence, Survival and Prevalence Statistics: Methodology Report*. Northern Ireland Cancer Registry Accessed February 4, 2025. https://www.qub.ac.uk/research-centres/nicr/FileStore/OfficialStatistics1993-2022/November24release/Methodology_report.pdf

2. Urban - Rural Classification. Northern Ireland Statistics and Research Agency. Accessed December 28, 2022. https://www.nisra.gov.uk/support/geography/urban-rural-classification

3. Northern Ireland Statistics and Research Agency (NISRA). Northern Ireland Multiple Deprivation Measure 2017 (NIMDM2017). Northern Ireland Statistics and Research Agency. 2017. Accessed August 22, 2024. https://www.nisra.gov.uk/statistics/deprivation/northern-ireland-multiple-deprivation-measure-2017-nimdm2017

4. O'Neill C, Donnelly DW, Harbinson M, et al. Survival of cancer patients with pre-existing heart disease. *BMC Cancer*. 2022;22(1):847. doi:10.1186/s12885-022-09944-z

Supplementary Appendix 2. Regression Results

Although we present full regression results here at the reviewer's request, we discourage readers from interpreting the coefficients of variables other than “pre-existing cardiovascular disease (CVD)” to avoid what is known as “The Table 2 Fallacy”[1].

Table S2.1. Logistic regression results for the likelihood of receiving chemotherapy among cancer patients (n=81338)

	Beta	SE	OR	Lower 95%CI	Upper 95%CI
**Intercept**	1.79	0.04	5.98	5.49	6.53
**Pre-existing CVD**	-0.36	0.03	0.70	0.67	0.73
**Age**	-0.04	0.00	0.96	0.96	0.96
**Sex (Male)**	-0.23	0.02	0.79	0.77	0.82
**Affluence**	0.01	0.00	1.01	1.00	1.01
**Region (Rural)**	0.03	0.02	1.03	0.99	1.07
**Cerebrovascular Disease**	-0.59	0.07	0.56	0.48	0.64
**Chronic Pulmonary Disease**	-0.23	0.03	0.80	0.75	0.85
**Hypertension**	-0.02	0.02	0.98	0.93	1.02
**Diabetes**	-0.18	0.04	0.83	0.77	0.90
**Liver Disease**	-0.34	0.08	0.71	0.61	0.83
**Renal Disease**	-0.39	0.06	0.68	0.60	0.77
**Peptic Ulcer**	0.08	0.07	1.08	0.94	1.24
**Anaemia**	0.05	0.05	1.05	0.95	1.16
**Neurodegenerative Disorders**	-0.82	0.06	0.44	0.39	0.50
**Rheumatoid Disorders**	-0.06	0.07	0.94	0.82	1.09

[1] Westreich D, Greenland S. The Table 2 Fallacy: Presenting and Interpreting Confounder and Modifier Coefficients. *Am J Epidemiol*. 2013;177(4):292-298. doi:10.1093/aje/kws412

Table S2.2. Logistic regression results for the likelihood of receiving hormone therapy among cancer patients (n= 23647) [a]

	Beta	SE	OR	Lower 95%CI	Upper 95%CI
**Intercept**	-0.12	0.08	0.88	0.75	1.03
**Pre-existing CVD**	0.02	0.04	1.02	0.94	1.11
**Age**	0.01	0.00	1.01	1.01	1.02
**Sex (Male)**	-0.30	0.03	0.74	0.70	0.79
**Affluence**	0.01	0.00	1.01	1.00	1.02
**Region (Rural)**	-0.07	0.03	0.94	0.88	0.99
**Cerebrovascular Disease**	-0.09	0.10	0.91	0.76	1.10
**Chronic Pulmonary Disease**	0.00	0.06	1.00	0.89	1.12
**Hypertension**	-0.21	0.04	0.81	0.75	0.87
**Diabetes**	-0.17	0.06	0.84	0.75	0.95
**Liver Disease**	0.00	0.19	1.00	0.69	1.46
**Renal Disease**	-0.18	0.10	0.83	0.69	1.01
**Peptic Ulcer**	0.28	0.14	1.32	1.01	1.72
**Anaemia**	-0.26	0.11	0.77	0.62	0.96
**Neurodegenerative Disorders**	-0.24	0.09	0.79	0.66	0.95
**Rheumatoid Disorders**	-0.09	0.13	0.91	0.71	1.18

[a] *Patients diagnosed with breast, prostate or uterine cancers between 2010 and 2019 are included.*

Table S2.3. Logistic regression results for the likelihood of receiving radiotherapy among cancer patients (n= 16041) [a]

	Beta	SE	OR	Lower 95%CI	Upper 95%CI
**Intercept**	0.26	0.09	1.30	1.09	1.55
**Pre-existing CVD**	-0.32	0.05	0.72	0.66	0.79
**Age**	-0.01	0.00	0.99	0.98	0.99
**Sex (Male)**	-0.25	0.04	0.78	0.72	0.83
**Affluence**	0.01	0.01	1.01	1.00	1.02
**Region (Rural)**	0.01	0.04	1.01	0.94	1.09
**Cerebrovascular Disease**	-0.20	0.11	0.82	0.66	1.02
**Chronic Pulmonary Disease**	-0.09	0.06	0.91	0.82	1.02
**Hypertension**	-0.15	0.05	0.86	0.79	0.95
**Diabetes**	-0.14	0.07	0.87	0.76	0.99
**Liver Disease**	-0.28	0.13	0.76	0.59	0.97
**Renal Disease**	-0.54	0.11	0.58	0.47	0.72
**Peptic Ulcer**	-0.01	0.14	0.99	0.76	1.29
**Anaemia**	-0.70	0.10	0.50	0.41	0.61
**Neurodegenerative Disorders**	-0.42	0.10	0.66	0.54	0.80
**Rheumatoid Disorders**	-0.23	0.13	0.80	0.62	1.02

[a] *Patients diagnosed between 2018 and 2019 are included.*

Table S2.4. Logistic regression results for the likelihood of receiving surgery among cancer patients (n=81338)

	Beta	SE	OR	Lower 95%CI	Upper 95%CI
**Intercept**	2.75	0.04	15.63	14.32	17.06
**Pre-existing CVD**	-0.26	0.02	0.77	0.74	0.80
**Age**	-0.03	0.00	0.97	0.97	0.97
**Sex (Male)**	-0.71	0.01	0.49	0.48	0.51
**Affluence**	0.02	0.00	1.02	1.01	1.02
**Region (Rural)**	0.06	0.02	1.06	1.02	1.09
**Cerebrovascular Disease**	-0.41	0.05	0.66	0.61	0.72
**Chronic Pulmonary Disease**	-0.39	0.03	0.68	0.64	0.71
**Hypertension**	0.06	0.02	1.06	1.02	1.10
**Diabetes**	-0.01	0.03	0.99	0.93	1.04
**Liver Disease**	-0.37	0.06	0.69	0.61	0.78
**Renal Disease**	-0.19	0.04	0.83	0.76	0.90
**Peptic Ulcer**	-0.05	0.06	0.96	0.85	1.07
**Anaemia**	0.12	0.04	1.13	1.04	1.23
**Neurodegenerative Disorders**	-0.62	0.04	0.54	0.49	0.58
**Rheumatoid Disorders**	-0.19	0.06	0.83	0.74	0.93

Table S2.5. Logistic regression results for the likelihood of receiving any treatment among cancer patients (n=81338)

	Beta	SE	OR	Lower 95%CI	Upper 95%CI
**Intercept**	3.64	0.05	38.12	34.34	42.32
**Pre-existing CVD**	-0.36	0.02	0.70	0.67	0.73
**Age**	-0.04	0.00	0.96	0.96	0.96
**Sex (Male)**	-0.34	0.02	0.71	0.69	0.73
**Affluence**	0.03	0.00	1.03	1.02	1.04
**Region (Rural)**	0.10	0.02	1.11	1.07	1.15
**Cerebrovascular Disease**	-0.46	0.04	0.63	0.58	0.69
**Chronic Pulmonary Disease**	-0.43	0.03	0.65	0.62	0.69
**Hypertension**	0.05	0.02	1.05	1.01	1.09
**Diabetes**	-0.14	0.03	0.87	0.83	0.92
**Liver Disease**	-0.59	0.06	0.55	0.49	0.62
**Renal Disease**	-0.31	0.04	0.73	0.68	0.79
**Peptic Ulcer**	-0.07	0.06	0.94	0.83	1.05
**Anaemia**	-0.04	0.04	0.96	0.88	1.04
**Neurodegenerative Disorders**	-0.82	0.04	0.44	0.41	0.48
**Rheumatoid Disorders**	-0.21	0.06	0.81	0.73	0.91

Table S2.6. The Cox model of the time to cancer treatment since the cancer diagnosis. (n=81338)

	Coef.	SE	HR	Lower 95%CI	Upper 95%CI
**Pre-existing CVD**	-0.15	0.01	0.86	0.84	0.88
**Age**	-0.01	0.00	0.99	0.99	0.99
**Sex (Male)**	-0.26	0.01	0.77	0.76	0.79
**Affluence**	0.01	0.00	1.01	1.01	1.01
**Region (Rural)**	0.04	0.01	1.04	1.02	1.06
**Cerebrovascular Disease**	-0.16	0.03	0.85	0.81	0.90
**Chronic Pulmonary Disease**	-0.16	0.02	0.86	0.83	0.89
**Hypertension**	0.09	0.01	1.10	1.07	1.12
**Diabetes**	-0.01	0.02	0.99	0.96	1.03
**Liver Disease**	-0.09	0.05	0.91	0.82	1.01
**Renal Disease**	-0.06	0.03	0.94	0.89	0.99
**Peptic Ulcer**	0.02	0.05	1.02	0.93	1.11
**Anaemia**	0.05	0.03	1.05	0.98	1.12
**Neurodegenerative Disorders**	-0.23	0.03	0.80	0.75	0.85
**Rheumatoid Disorders**	-0.05	0.04	0.95	0.88	1.03

Supplementary Appendix 3. Subgroup analyses figures

Each adjusted odds ratio is estimated by a separate logistic regression model which is adjusted for confounders including age, sex, deprivation, rurality and other comorbidities. The number of patients with given Cancer-CVD combinations is provided in parentheses. Red corresponds to a negative effect (less likely to have treatment) and blue to a positive (more likely to have treatment), with darker shades indicating greater effect size. Values with confidence intervals containing null are omitted here for visual clarity. A detailed table of estimates with corresponding confidence intervals can be found at osf.io/vgzq5.

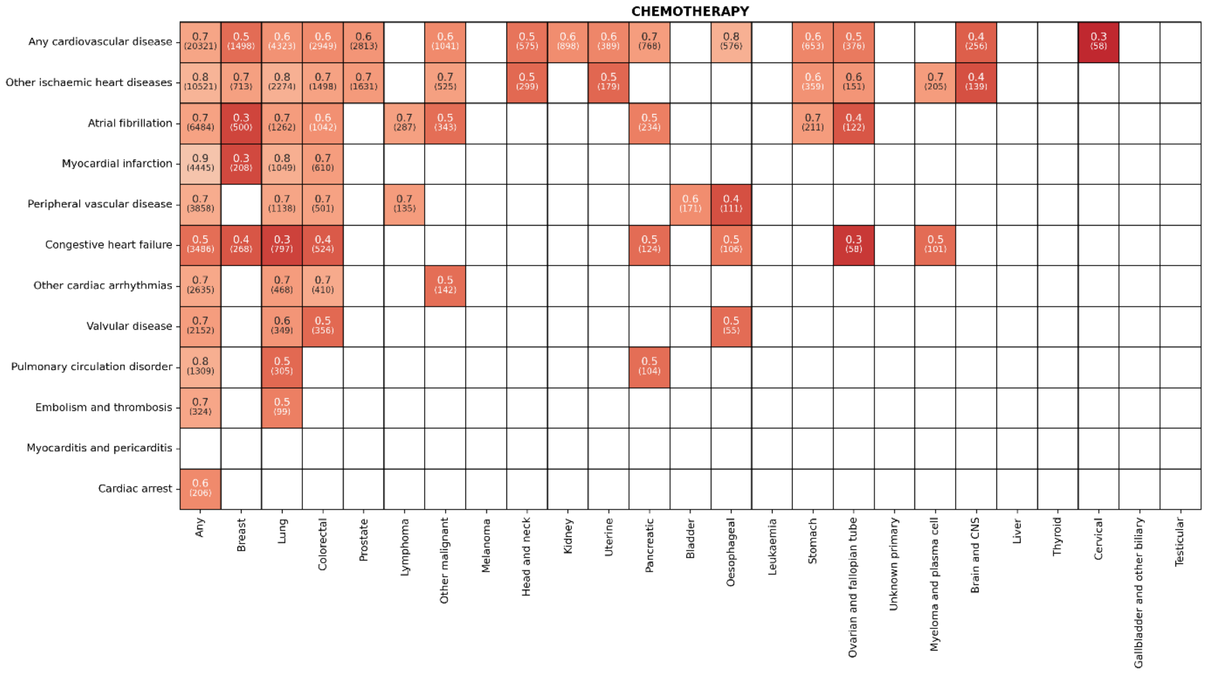

Figure S3.1. Adjusted odds ratios for receiving chemotherapy for cancer patients with pre-existing CVD by tumour sites and cardiovascular disease types.

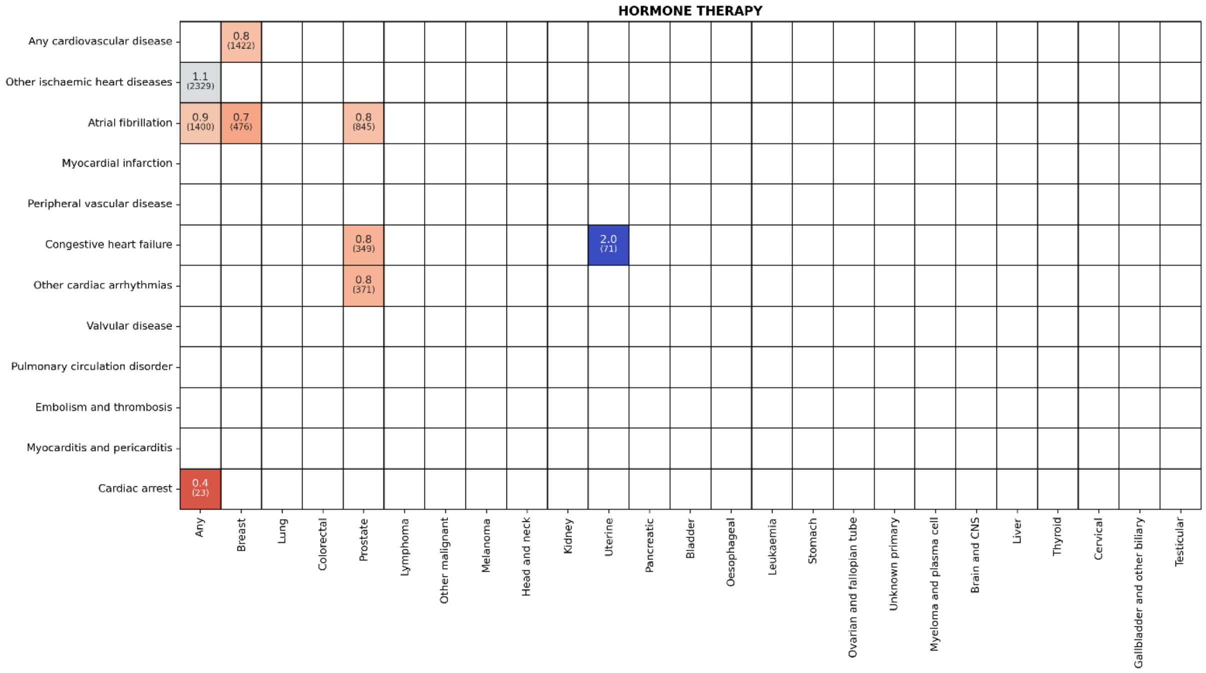

Figure S3.2. Adjusted odds ratios for receiving hormonal therapy for cancer patients with pre-existing CVD by tumour sites and cardiovascular disease types.

Only patients diagnosed with breast, prostate or uterine cancers between 2010 and 2019 are included.

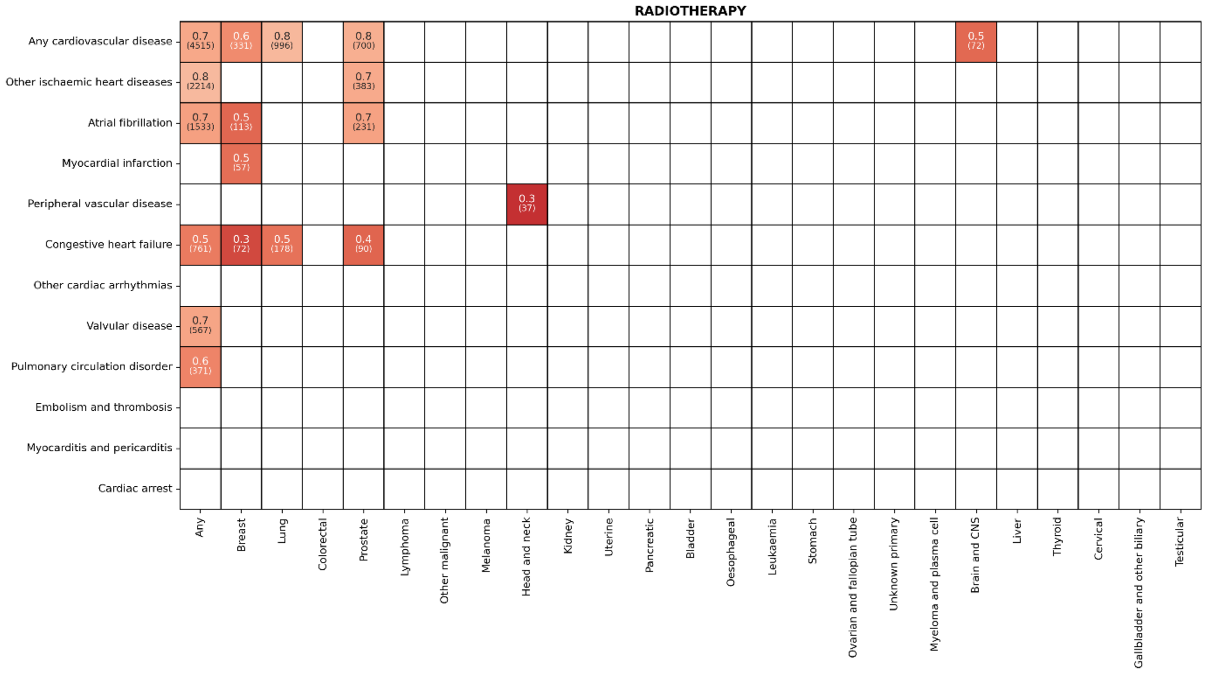

Figure S3.3. Adjusted odds ratios for receiving radiotherapy for cancer patients with pre-existing CVD by tumour sites and cardiovascular disease types.

Only patients diagnosed between 2018 and 2019 are included.

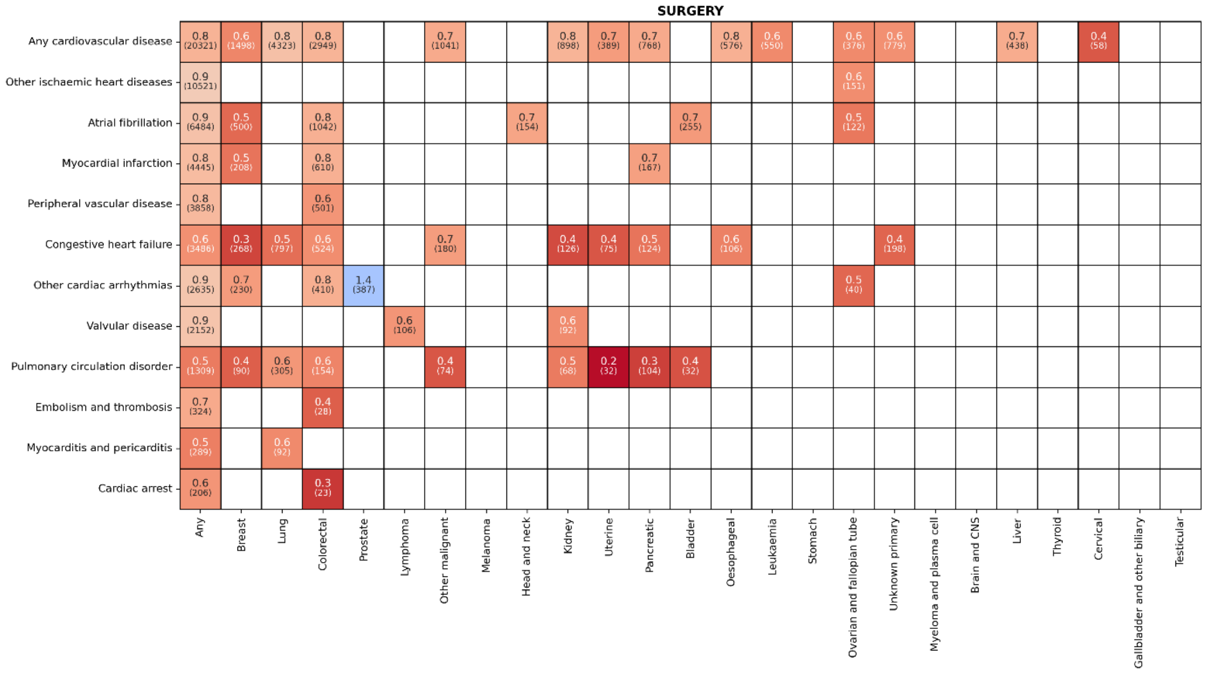

Figure S3.4. Adjusted odds ratios for receiving surgery for cancer patients with pre-existing CVD by tumour sites and cardiovascular disease types.

Supplemental Appendix 4. Sensitivity analyses

Table S4.1. Logistic regression model of the association between pre-existing CVD and the likelihood of receiving any cancer treatment (n=81341)

	Beta	SE	OR	Lower 95%CI	Upper 95%CI
**Intercept**	1.07	0.01	2.91	2.86	2.97
**Pre-existing CVD**	-0.85	0.02	0.43	0.41	0.44

Table S4.2. Logistic regression model of the association between pre-existing CVD and the likelihood of receiving any cancer treatment, adjusting for only demographic and socioeconomic factors (n=81338)

	**Beta**	**SE**	**OR**	**Lower 95%CI**	**Upper 95%CI**
**Intercept**	3.62	0.05	37.19	33.55	41.23
**Pre-existing CVD**	-0.44	0.02	0.64	0.62	0.67
**Age**	-0.04	0.00	0.96	0.96	0.96
**Sex (Male)**	-0.33	0.02	0.72	0.70	0.75
**Affluence**	0.04	0.00	1.04	1.03	1.04
**Region (Rural)**	0.12	0.02	1.12	1.09	1.16

Table S4.3. Logistic regression model of the association between pre-existing CVD and the likelihood of receiving any cancer treatment, adjusting for all observed confounders. (n=81338)

	Beta	SE	OR	Lower 95%CI	Upper 95%CI
**Intercept**	3.64	0.05	38.12	34.34	42.32
**Pre-existing CVD**	-0.36	0.02	0.70	0.67	0.73
**Age**	-0.04	0.00	0.96	0.96	0.96
**Sex (Male)**	-0.34	0.02	0.71	0.69	0.73
**Affluence**	0.03	0.00	1.03	1.02	1.04
**Region (Rural)**	0.10	0.02	1.11	1.07	1.15
**Cerebrovascular Disease**	-0.46	0.04	0.63	0.58	0.69
**Chronic Pulmonary Disease**	-0.43	0.03	0.65	0.62	0.69
**Hypertension**	0.05	0.02	1.05	1.01	1.09
**Diabetes**	-0.14	0.03	0.87	0.83	0.92
**Liver Disease**	-0.59	0.06	0.55	0.49	0.62
**Renal Disease**	-0.31	0.04	0.73	0.68	0.79
**Peptic Ulcer**	-0.07	0.06	0.94	0.83	1.05
**Anaemia**	-0.04	0.04	0.96	0.88	1.04
**Neurodegenerative Disorders**	-0.82	0.04	0.44	0.41	0.48
**Rheumatoid Disorders**	-0.21	0.06	0.81	0.73	0.91

Table S4.4. Logistic regression model of the association between pre-existing CVD on the likelihood of receiving any cancer treatment, adjusting for all observed confounders and tumour stage. (n=65124)

	Beta	SE	OR	Lower 95%CI	Upper 95%CI
**Intercept**	3.99	0.07	54.21	47.61	61.74
**Pre-existing CVD**	-0.28	0.02	0.76	0.72	0.79
**Age**	-0.03	0.00	0.97	0.97	0.97
**Sex (Male)**	-0.46	0.02	0.63	0.61	0.66
**Affluence**	0.03	0.00	1.03	1.03	1.04
**Region (Rural)**	0.08	0.02	1.08	1.04	1.13
**Cerebrovascular Disease**	-0.44	0.05	0.65	0.58	0.71
**Chronic Pulmonary Disease**	-0.48	0.03	0.62	0.58	0.65
**Hypertension**	0.02	0.02	1.02	0.97	1.07
**Diabetes**	-0.12	0.03	0.89	0.83	0.95
**Liver Disease**	-0.41	0.08	0.66	0.57	0.77
**Renal Disease**	-0.23	0.05	0.80	0.72	0.88
**Peptic Ulcer**	0.04	0.07	1.04	0.90	1.21
**Anaemia**	0.08	0.05	1.09	0.98	1.21
**Neurodegenerative Disorders**	-0.63	0.05	0.53	0.48	0.59
**Rheumatoid Disorders**	-0.17	0.07	0.85	0.74	0.97
**Tumour Stage ***	-0.24	0.01	0.79	0.78	0.80

** The tumour stage is a mediator and not a confounder in the relationship between pre-existing CVD and cancer treatment receipt (as seen in Figure 1 in the main text). Therefore it should not have been adjusted for this particular analysis. Here it is included in the model at the reviewer's request.*

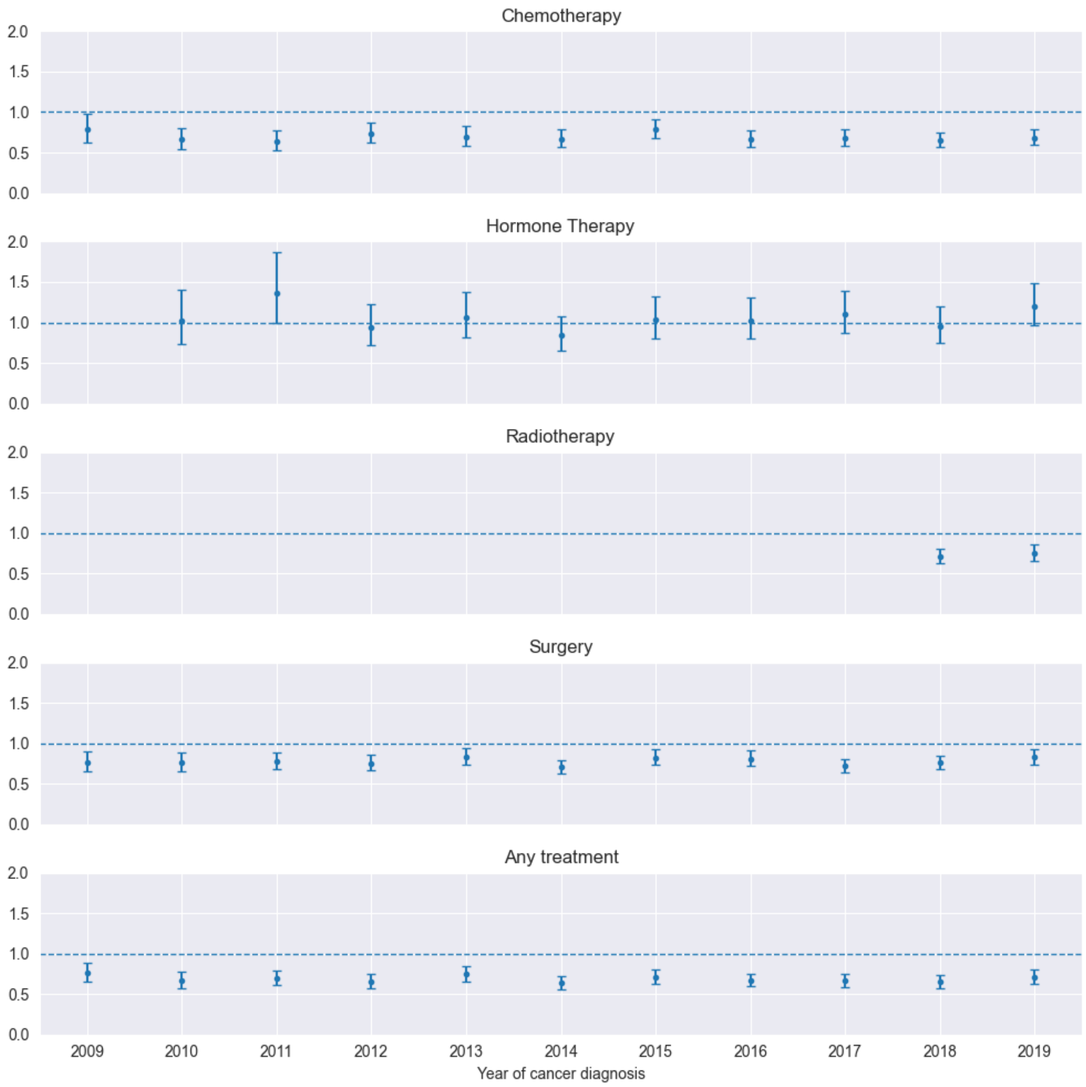

Figure S4.1. Adjusted odds ratio of receiving cancer treatment for cancer patients with pre-existing CVD by treatment modalities and year of cancer diagnosis.

Each adjusted odds ratio is estimated by a separate logistic regression model, which is adjusted for confounders including age, sex, deprivation, rurality and other comorbidities. Error bars show 95% confidence intervals.
